# Equipment maintenance and repair

**Published:** 2010-09

**Authors:** DS Walia, Jane Huria, Ismael Cordero

**Affiliations:** Director of Clinical Services: Eye Unit, PCEA Kikuyu Hospital, PO Box 45-00902, Kikuyu, Kenya. Email: dcseye@pceakikuyuhospital.org; Instrument Repair Technician, Eye Unit, PCEA Kikuyu Hospital.; Senior Clinical Engineer, ORBIS International, 520 8th Ave, llth Floor, New York, NY 10018, USA.

**Figure:**
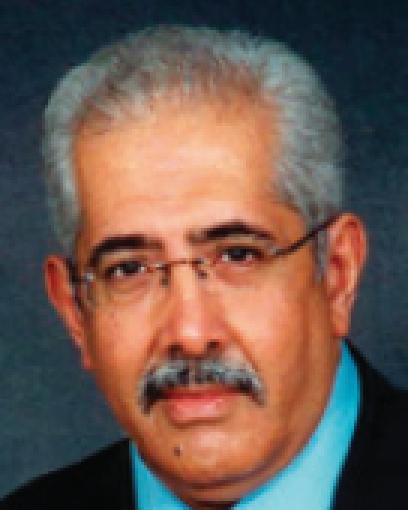


**Figure:**
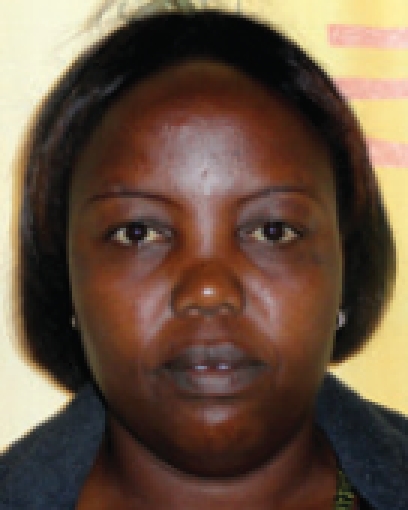


**Figure:**
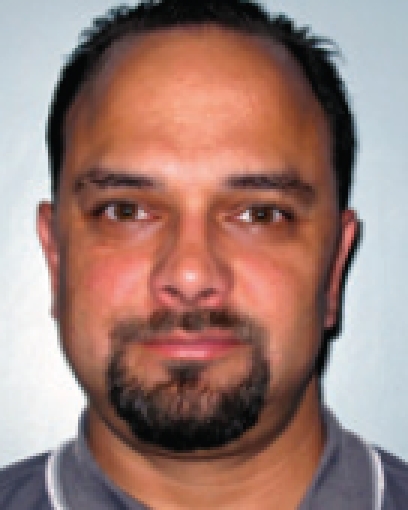


The repair and maintenance of ophthalmic equipment, including surgical instruments and diagnostic devices, can be compared to the maintenance of a motor vehicle, something many of us understand well.

If you had a car, would you drive it until the fuel runs out or until a tyre punctures, and then abandon it to buy a new car? Of course not. However, many eye care units purchase (or receive as a donation) expensive and delicate equipment which, because of poor maintenance, ends up breaking down. If there is not a system in place to report breakdowns and to plan or carry out repairs, equipment can remain unusable for long periods of time. Sometimes, this equipment ends up being dumped. (Figure 1).

**Figure 1:**
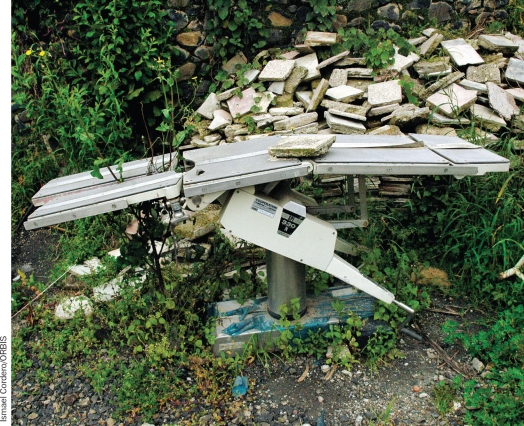
An operating table lies abandoned outside a hospital in a low-income country.

Ismael Cordero/ORBIS

Good maintenance habits and an effective repair system will minimise the amount of time equipment is unusable.

## Who does what?

In our experience, approximately one-third of problems reported with ophthalmic equipment arise from problems caused by the user, one-third from easy-to-solve technical problems (such as a blown bulb or fuse, or a loose power cord), and only one-third require more serious fault-finding procedures and special knowledge of the equipment. Equipment users therefore have a significant role to play in the everyday care and maintenance of equipment.

Usually, a well-balanced mix of user, in-house, and outsourced maintenance and repair leads to the best results - both technical and financial - in settings with limited resources. If it is not feasible for an eye care unit to have an in-house equipment maintenance and repair team, you may consider sharing such a service among several units. Depending on the equipment, you may have a service contract with the vendor or manufacturer, who will be responsible for more complex maintenance and repairs. These will be carried out by specialised maintenance and repair personnel, either employed by the vendor or manufacturer, or working as independent maintenance contractors. Whatever system your eye unit has in place, the maintenance and repair of equipment should be centrally managed. The person responsible (the ‘equipment person’) will assign tasks, keep maintenance and repair records, design maintenance schedules, and arrange the necessary training of staff.

Sometimes, maintenance or repair support may be required from vendors and other external maintenance contractors. In all of these instances, it is important that a designated person at the eye care unit is monitoring the responsiveness, quality, and cost of the service provided.

**‘The maintenance and repair of equipment should be centrally managed’**

## Preventative maintenance

Preventative maintenance prevents breakdowns and ensures that equipment is operational and safe to use. It also guarantees the accuracy and reliability of equipment (that the autoclave sterilises properly and the keratometer readings are correct, for example) and saves money: it can reduce the running costs of equipment and is cheaper than repairs following a breakdown.

Preventative maintenance consists of a number of tasks of varying technical complexity, carried out by different groups of people.

- **Equipment users,** including clinic and operating theatre staff, can be trained to perform many of the simple care and maintenance duties that need to be done on a regular basis, such as dusting, cleaning, lubricating, protecting, and checking equipment, including safety checks.
- Other maintenance tasks can be performed by an in-house or shared **maintenance and repair team** that has been given additional training. These include tasks such as cleaning a microscope lens, replacing an electronic component, performing a mechanical adjustment, or any other action that requires mechanical skills and/or a knowledge of electronics.
- More complex work has to be done by **specialised maintenance and repair personnel** contracted or employed by the vendor or manufacturer.

## Preventative maintenance schedules

It is important to have a schedule for preventative maintenance of each item of equipment. This consists of a **timetable** stating when (and how frequently) maintenance should be done, and a **list of maintenance activities** for each item. These schedules should provide simple guidelines for all types of equipment, covering the tasks to be undertaken in the following areas:

- Care and cleaning
- Safety checks
- Functional and performance checks
- Maintenance tasks (changing bulbs, lubricating moving parts, etc.)

The best source of this information is usually the manufacturer's user and/or service manual.

Schedules need to be developed separately for both users and maintainers. For example, users can perform checks and basic maintenance tasks on a daily basis, whereas the maintenance team can set aside a specific day of the week or month to carry out regular maintenance tasks. More sophisticated maintenance tasks, such as those which need to be carried out by service agents, should be scheduled for a specific day or week in the year.

It is helpful to display maintenance schedules for users on or near the equipment they refer to; this can serve as a useful daily reminder of the tasks that should be performed.

## Repair

Repair means responding to the breakdown of equipment and undertaking work to correct the problem in order to return the equipment to a working condition.

Before equipment can be repaired, you need to be aware that there is a problem! Therefore, there should be a clearly understood **system for reporting faults and breakdowns** and equipment users should be encouraged to report faults and breakdowns as soon as possible. If there is no back-up equipment, a breakdown will mean that the service the equipment was providing will come to a halt.

**Simple repairs** can be done by the in-house or external **maintenance and repair team**. If the equipment is repaired where it is used, it is important that the team is trained to work safely and that they don't create hazards for patients or staff.

**More complex repairs** will be carried out by **specialised maintenance personnel**; they might come to the eye care unit or you may have to send the equipment to them for repairs.

In all these situations, it is important to keep equipment users informed of how long their equipment will be unavailable.

Some items of equipment will be found to be damaged beyond repair. For others, spare parts may no longer be available as the equipment has become outdated. These will have reached the end of their lives and must be taken out of service (decommissioned or retired) and be replaced if the service they provide is to continue. Equipment that is being decommissioned should be disposed of safely and according to proper disposal procedures. Remember to update your records accordingly.

**‘Plan for maintenance when you purchase the equipment’**

## Record-keeping

In order for an eye care unit to manage its equipment effectively, it needs good maintenance and repair records. It is very difficult to manage the unknown!

A **central maintenance and repair record** will help you to keep track of the maintenance and repair work done. Ideally, this system should correspond to the eye unit's equipment inventory (mentioned on page 34); this means that you will have maintenance and repair records for each of the items listed in the inventory.

### Record-keeping for maintenance

The preventative maintenance schedule for users can be accompanied by a weekly or monthly ‘tick sheet’ near the item of equipment, with a space for each day so that users can date and sign it, thereby showing that they have carried out the required tasks. This may include a space for users to indicate what spare parts, such as bulbs, were used. On a regular basis, the list of spare parts used should be noted in the central maintenance and repair record so that more spare parts can be ordered.

The central maintenance and repair record can be used to keep track of all other maintenance, including maintenance done by the in-house team, by vendors, or by service agents. The information captured should include the date, the equipment reference number, what was done, who did the work, and when next maintenance is due.

**Figure 2:**
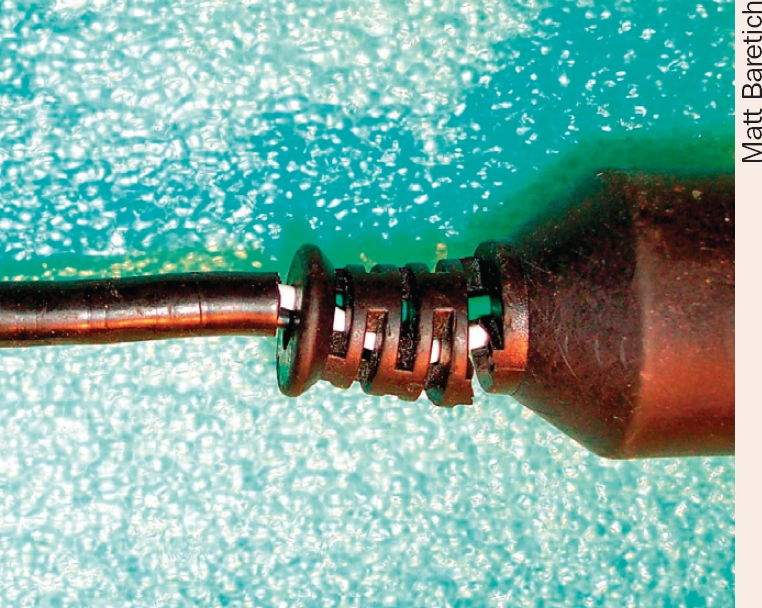
One way of keeping track of regular maintenance tasks is to affix a tag to serviced or maintained equipment. This information should be reflected in the central maintenance record.

Ismael Cordero/ORBIS

### Record-keeping for repair

Table 1 shows what information about repairs should be recorded in the central maintenance and repair record, and what useful information this can provide.

In addition to the practical benefits of a central maintenance and repair system, it also provides eye care unit administrators and the equipment maintenance team with valuable information and proof that they can use to ask for more resources.

**Table 1:** Record-keeping for repair

What should be recorded	This provides information about…
The details of repair work done on each machine (including cause/suspected cause, and who carried out the repair)	The history of each machine Common problems
The spare parts and materials used	The parts most frequently used What needs to be re-ordered
The date equipment has broken down, and the date it is repaired.	What still needs to be repaired (which allows you to prioritise the next week's tasks) The duration equipment is not in use (down-time)
The causes of any delays	What the most common causes of delays are (skill, labour, spare parts, transport, bureaucratic delays, money) and what additional resources may be needed to complete work on time

## Budgeting for maintenance and repair

When we purchase a motor vehicle, we understand that we will have recurring costs for maintenance, theft and accident insurance, cleaning, parking, etc. The same is true for ophthalmic equipment since it costs money to operate and to maintain during its life cycle.

On average, the original purchase cost only makes up about twenty per cent of the entire life cycle cost of the equipment. As a general rule, you should budget anywhere from 3% to 6% of the equipment purchase cost per year for each device to cover consumables, parts, maintenance, and user training.

Plan for maintenance **when you purchase the equipment** and ensure that you buy the necessary accessories (including voltage stabilisers/surge protectors and uninterrupted power supply units) as well as enough spare parts (bulbs, fuses, and so on) to last for at least a year.

In conclusion, adopting practical and workable systems to manage eye care equipment, as suggested in this article, will help you to get the most use out of the equipment you have. With equipment, prevention is usually better than cure! It is also good practice to keep learning and to stay open to new ideas. Communicate with colleagues in other eye units, whether locally or through the internet, about the challenges you face and share with them the solutions you have found.

**TOP TIPS Equipment care**

**Tools**

Where possible, avoid using hammers, pliers, and files: these usually cause irreparable damage. Use the right tool to tighten screws and other parts.

**Lubrication**

- **Petroleum jelly** (Vaseline) or white grease is good for lubricating parts that have gears or sliding surfaces. It is clear and less likely than regular grease to leave stains on hands and clothing.
- **Silicone spray** is useful for sliding plastic or nylon parts. Take care not to spill any on the floor as it is very slippery and hard to remove.
- **Graphite** can be used as a dry lubricant (or as a paste) for moving or sliding parts.
- Avoid **excessive use of oil** as it is messy and collects dust.

**Foot pedals**

These frequently become wet when floors are being cleaned, especially in the operating theatre. Moisture seeps into the electrical components of the foot pedal which with time will stop working. Always place any foot pedal off the floor when mopping.

**Wires and optical fibres**

Wires and optical fibres from equipment such as laser machines, vitrectors, indirect ophthalmoscopes, etc. have very delicate interiors and/or optical components within them, and some of them may carry power or electricity and heat up with use. Staff should know how to handle and fold them properly in order to prevent damage. In situations where wires and fibres have to lie on the floor, they should not be walked on or run over with heavy items such as trolleys or other wheeled furniture and equipment.

**Lenses**

Protect lenses from dust by always covering optical equipment when not in use.

Humidity, or liquid spilled on instruments, can cause fungal growth (mould) on lenses. Many producers of optical equipment supply sachets of silica gel drying agents or fungicidal (anti-mould) pellets that you can place inside the dust cover. You can also use a dehumidifier to keep the air in the room dry.

**Electrical connections**

**Figure:**
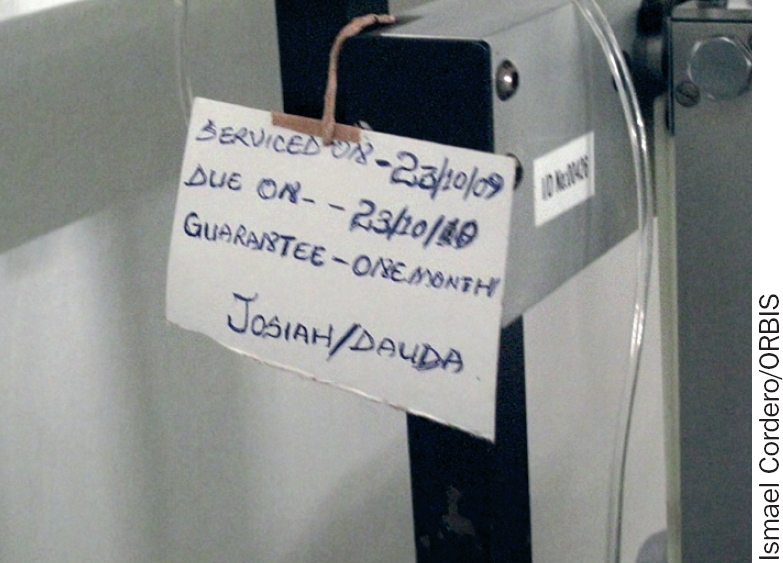
Pulling on the cord instead of on the plug can damage the wires.

Matt Baretich

Check that equipment is plugged in properly and that the cord is not in danger of shorting (for example, if it is exposed to water or steam) or in danger of being cut.

Handle plugs (or connectors) with care. A break in a wire inside the plug casing is hard to find and causes equipment to work intermittently. This can result from bending the wire at the same place or unplugging from the socket by pulling on the cord.

**Power sources**

Expensive and delicate equipment such as bench-top autoclaves, lasers, microscopes, slit lamps, and vitrectors can be severely damaged by sudden surges in electricity. Using a **voltage stabiliser** or **regulator** will protect equipment against damage and will generally also prolong the life of equipment. We recommend good quality units which monitor the mains voltage continuously. Look for units which will stabilise the output to ensure the voltage reaching your equipment remains constant at 230V (±6%). If the input voltage falls below 142V or rises above 295V, the stabiliser will automatically disconnect the output. Stabilisers of poorer quality may be overwhelmed by large fluctuations, which will then damage any connected equipment.

The use of an **uninterrupted power supply** (UPS) unit is strongly recommended for equipment such A-scans, visual field analysers, fundus cameras, lasers, and operating microscopes. This ensures continuous operation of the equipment and less inconvenience for both surgeons and patients when there are power failures. We recommend using a UPS that is also able to act as a voltage stabiliser and protect the equipment against power surges. The type of UPS and its power output requirements can easily be determined by a qualified electrician. Equipment suppliers may also be able to give these details.

**Using equipment for outreach**

Equipment and instruments that are transported for outreach work need to be packed and carried with special precautions so they are not damaged in transit.

Heavier equipment such as operating microscopes should be carefully dismantled and packed in damage-proof containers. Specially designed microscopes for mobile use are available with proper packing containers for safe transport.

Sufficient stock of light bulbs and fuses should be carried together with a set of screw drivers and other basic tools. Staff travelling with outreach programmes should know how to pack, set up, and dismantle the equipment.

With thanks to Ingrid Mason, Neil Murray, Kola Ogundimu, Sam Powdrill, Tony Walia, and Ismael Cordero.

**What YOU can do to look after your equipment**

Care and maintenance of equipment is everyone's responsibility; unusable equipment affects the quality of care we can offer our patients and makes our work more stressful.

Whatever your role in the eye care team, there is a lot you can do to look after and prolong the life of the equipment you use.

1. **Learn as much as you can about the equipment you use**
  - Find out where the manual is kept-and make time to read it. If possible, keep the manuals close to the equipment.
  - Make sure you get every issue of the *Community Eye Health Journal* over the next three to four years - we are publishing a new series on equipment care, maintenance, and repair which will have practical tips and guidance on the most used items (see the first instalment on page 37).
  - Look at ‘Useful resources’ (page 36) for additional sources of information.
2. **Check equipment before use (or at least once a week)**
  - Inspect equipment for any sign of damage or parts that may need repair or replacement, and lubricate as necessary (according to instructions).
  - Check that equipment is plugged into the voltage stabiliser or uninterrupted power supply, where these are required.
3. **Tell someone if there is a problem**
  - As a user, it is your responsibility to report any problems. You will most likely be the first person to know that something is not working as it should.
  - Don't assume that someone else will report a fault-what if everyone thinks that someone else will report it?
  - It may sound obvious, but a repair can't be attended to if nobody knows there is a problem. The longer you take to report it, the longer before the repair will take place.
  - Don't wait for equipment to break down before reporting a fault. Even a small change in how the equipment moves or how it responds could indicate that something has gone wrong or that a part needs to be replaced soon. If left unchecked, a more serious fault may occur, which will be more expensive and time-consuming to repair.
4. **Clean equipment after use (or at least once a day) and lubricate when necessary**
  - Dust and then clean equipment, including optical components, with the appropriate cleaning agents and solutions. Lubricate moving equipment as often as indicated. Always follow the instructions.
5. **Protect equipment when not in use**
  - Store equipment in a dry, clean environment where it is not in danger of falling and breaking.
  - Place plastic dust covers on larger equipment like slit lamps in order to prevent damage to the optics and other delicate components. If cloth is used, ensure it is heavy and non-porous, or else dust will get through.
  - When transporting equipment, pack items securely and handle with care.

